# Fluorescence optical imaging feature selection with machine learning for differential diagnosis of selected rheumatic diseases

**DOI:** 10.3389/fmed.2023.1228833

**Published:** 2023-08-21

**Authors:** Felix Rothe, Jörn Berger, Pia Welker, Richard Fiebelkorn, Stefan Kupper, Denise Kiesel, Egbert Gedat, Sarah Ohrndorf

**Affiliations:** ^1^Telematics Research Group, Wildau Technical University of Applied Sciences, Wildau, Germany; ^2^Xiralite GmbH, Berlin, Germany; ^3^Institute of Functional Anatomy, Charité—Universitätsmedizin Berlin, Berlin, Germany; ^4^Department of Rheumatology and Clinical Immunology, Charité—Universitätsmedizin Berlin, Berlin, Germany

**Keywords:** rheumatoid arthritis, osteoarthritis, connective tissue diseases, gradient boosting machine, forward selection algorithm

## Abstract

**Background and objective:**

Accurate and fast diagnosis of rheumatic diseases affecting the hands is essential for further treatment decisions. Fluorescence optical imaging (FOI) visualizes inflammation-induced impaired microcirculation by increasing signal intensity, resulting in different image features. This analysis aimed to find specific image features in FOI that might be important for accurately diagnosing different rheumatic diseases.

**Patients and methods:**

FOI images of the hands of patients with different types of rheumatic diseases, such as rheumatoid arthritis (RA), osteoarthritis (OA), and connective tissue diseases (CTD), were assessed in a reading of 20 different image features in three phases of the contrast agent dynamics, yielding 60 different features for each patient. The readings were analyzed for mutual differential diagnosis of the three diseases (One-vs-One) and each disease in all data (One-vs-Rest). In the first step, statistical tools and machine-learning-based methods were applied to reveal the importance rankings of the features, that is, to find features that contribute most to the model-based classification. In the second step machine learning with a stepwise increasing number of features was applied, sequentially adding at each step the most crucial remaining feature to extract a minimized subset that yields the highest diagnostic accuracy.

**Results:**

In total, *n* = 605 FOI of both hands were analyzed (*n* = 235 with RA, *n* = 229 with OA, and *n* = 141 with CTD). All classification problems showed maximum accuracy with a reduced set of image features. For RA-vs.-OA, five features were needed for high accuracy. For RA-vs.-CTD ten, OA-vs.-CTD sixteen, RA-vs.-Rest five, OA-vs.-Rest eleven, and CTD-vs-Rest fifteen, features were needed, respectively. For all problems, the final importance ranking of the features with respect to the contrast agent dynamics was determined.

**Conclusions:**

With the presented investigations, the set of features in FOI examinations relevant to the differential diagnosis of the selected rheumatic diseases could be remarkably reduced, providing helpful information for the physician.

## 1. Introduction

Successful treatment of rheumatic diseases affecting the hands depends on an accurate and fast diagnosis in patients presenting with hand pain (1). Fluorescence optical imaging (FOI) has been used to study a number of rheumatic diseases, including, for example, rheumatoid arthritis (RA) (2–4), osteoarthritis (OA) (4, 5), connective tissue diseases (CTD) (6, 7), and others (8, 9). The results of FOI for the diagnosis of rheumatic diseases have been compared to MRI [3, 5, 9] and ultrasonography (2–5, 9, 10) with good agreement. Inflammatory changes in the microcirculation can be visualized using FOI. Until recently, the primary focus has been on signal enhancement in the joint regions of both hands and wrists (8). However, FOI examinations display more, less commonly used image features that may also be of diagnostic value. These include spots on the hand, distensions from the nail bed, the occurrence of Raynaud's syndrome in CTD, and others (6, 10). Eighteen different features found in FOI examinations have recently been presented (11).

When analyzing FOI images, it is important, on the one hand, to find specific features that contribute value to the correct diagnosis and, on the other hand, to focus on a minimal set of decision-making features in terms of feasibility. To achieve this, we used statistical and machine learning-based feature selection methods.

Machine learning algorithms are not explicitly programmed to approach a problem but extract knowledge about the solution from input data, e.g., solution-dependent patterns or associations (12). That is, complex non-linear interaction effects in the data are captured in an exploratory way without the need to explicitly specify these in the model definition (13). Due to these capabilities and the increasing availability of usable data, the utilization of machine learning in rheumatologic diagnostics and research is growing. A comprehensive review of the application of machine learning in rheumatology and a description of machine learning terms, algorithms, and workflows are available (12).

Feature selection denotes identifying features among the data that are important for solving the problem. Although feature selection is often conducted to improve the generalizability and performance of a predictive machine learning model, it can be used to extract only the most informative features and remove noisy, non-informative, irrelevant, and redundant features (14) and therefore help researchers understand the biological process(es) that underlie a disease (15). Therefore, feature selection is widely applied to medical problems (14, 16) and was applied to rheumatological problems with different kinds of data (12), including personal health records (17), genomics (18), and ultrasound (19). The aim of this analysis was to find specific image features for making accurate diagnoses of different rheumatic diseases affecting the hands.

## 2. Methods

### 2.1. Patients

FOI examinations were available in a database containing 3,690 patients with known clinical diagnoses. Three cohorts were compiled from the database, including patients with rheumatoid arthritis (RA), osteoarthritis (OA), or connective tissue diseases (CTD). The main criterion for the inclusion of patients in the cohorts was the known clinical diagnosis of the mentioned diseases made by a rheumatologist without knowledge of the FOI images. Additional criteria for inclusion in the RA cohort were a manifestation of Steinbrocker II-IV in the patients, which was examined by corresponding bone erosion in the X-ray images, which were also available in the database. The number of OA patients in the database was quite large, so to have comparable cohort sizes and not overload the reading, the OA cohort was constrained by random choice. For the CTD cohort, all patients were included, and no further exclusion criteria were applied. The images of the FOI examinations were not used for inclusion decisions.

### 2.2. FOI imaging

FOI examinations were conducted with the Xiralite (Berlin, Germany) X4 NIR-fluorescence optical imaging scanner. The hands of the patients were placed in the device, and an examination was started. After 10 s, an indocyanine green (ICG) fluorescence contrast agent was administered intravenously at a dose of 0.1 mg/kg body weight. The examinations took 6 min, with one image recorded per second, resulting in 360 images. FOI examinations were conducted at study sites at 10 resident rheumatologists' offices.

### 2.3. Readings

Image sequences were divided into three phases. Using specialized software, the reader first selected the end of phase 1 separately for the left and right hands. The end of phase 1 was characterized by the beginning of the apparent backflow of the dye from the nail bed area of fingers II–V. Phase 2 started thereafter and ended with frame 150. Phase 3 comprised all the following images to the end. Thereafter, the images of each phase were summed up and presented to the reader, resulting in three images per patient being assessed. The reader selected the observable image features from a list of all features or marked the phase image as featureless. The feature list comprised 20 features−15 described previously (11), including joint-related features (D, P, M, C, and O), finger-related features (r, R), nail features (a, I), the venous vessel feature (V), connective tissue features (E, B, and Y), and skin features (F, W). Five additional features (H, S, T, U, Z) were used in the presented reading. They are introduced in Table 1.

**Table 1:** Features used in the reading that are not yet described in the literature.

**Description**	**Acronym**	**Image**
Veins at the metacarpophalangeal joint	H	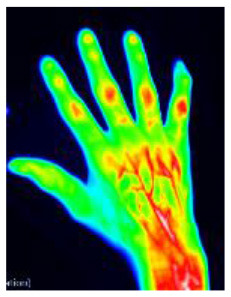
Starry sky pattern on the back of the hand	S	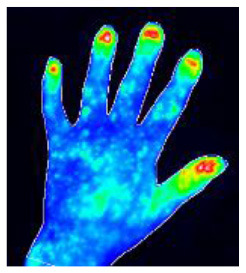
Streaky pattern	T	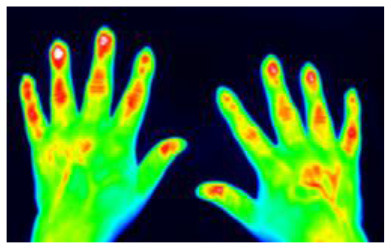
		
U- or o-shaped pattern around a fingernail	U	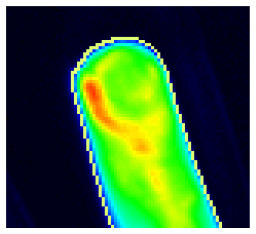
Spots in the nail bed	Z	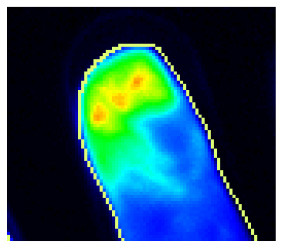

### 2.4. Data preprocessing

The clinical diagnosis was made independent of which patient's hands were affected. Thus, prior to further analysis, the features annotated for each hand separately were fused, removing the information on which hand a feature occurred.

The annotated features were collected in feature vectors, representing observations by a list of binary variables. With the named 20 examined features in three different phases, each observation initially consisted of 60 explanatory variables and one target variable representing a three-class classification problem (RA, OA, or CTD). For further analysis, this multinomial problem was transferred to a set of binomial classifications. This allowed for a more in-depth analysis of relevant disease-specific features and a differentiated diagnostic performance analysis. That is, three One-vs-Rest (OvR) and three One-vs-One (OvO) problems were evaluated. In the OvR problems, one diagnosis (positive class) was contrasted against the remaining two other diagnoses (fused to one negative class) (20). For OvO, each possible pair of classes was contrasted against each other (one diagnosis is considered negative, the other one positive) while ignoring the observations of the one remaining class (21). In summary, six problems were considered: RA-vs.-OA, OA-vs.-CTD, RA-vs.-CTD, RA-vs.-Rest, OA-vs.-Rest, and CTD-vs.-Rest.

As is common in machine-learning-based data analysis, the data were split randomly into a training set (90%) used for all further investigations and a test set (10% holdout set), which was retained from all analysis steps to evaluate the final results with an unbiased performance estimate (22).

### 2.5. Feature selection

**Figure 1:**
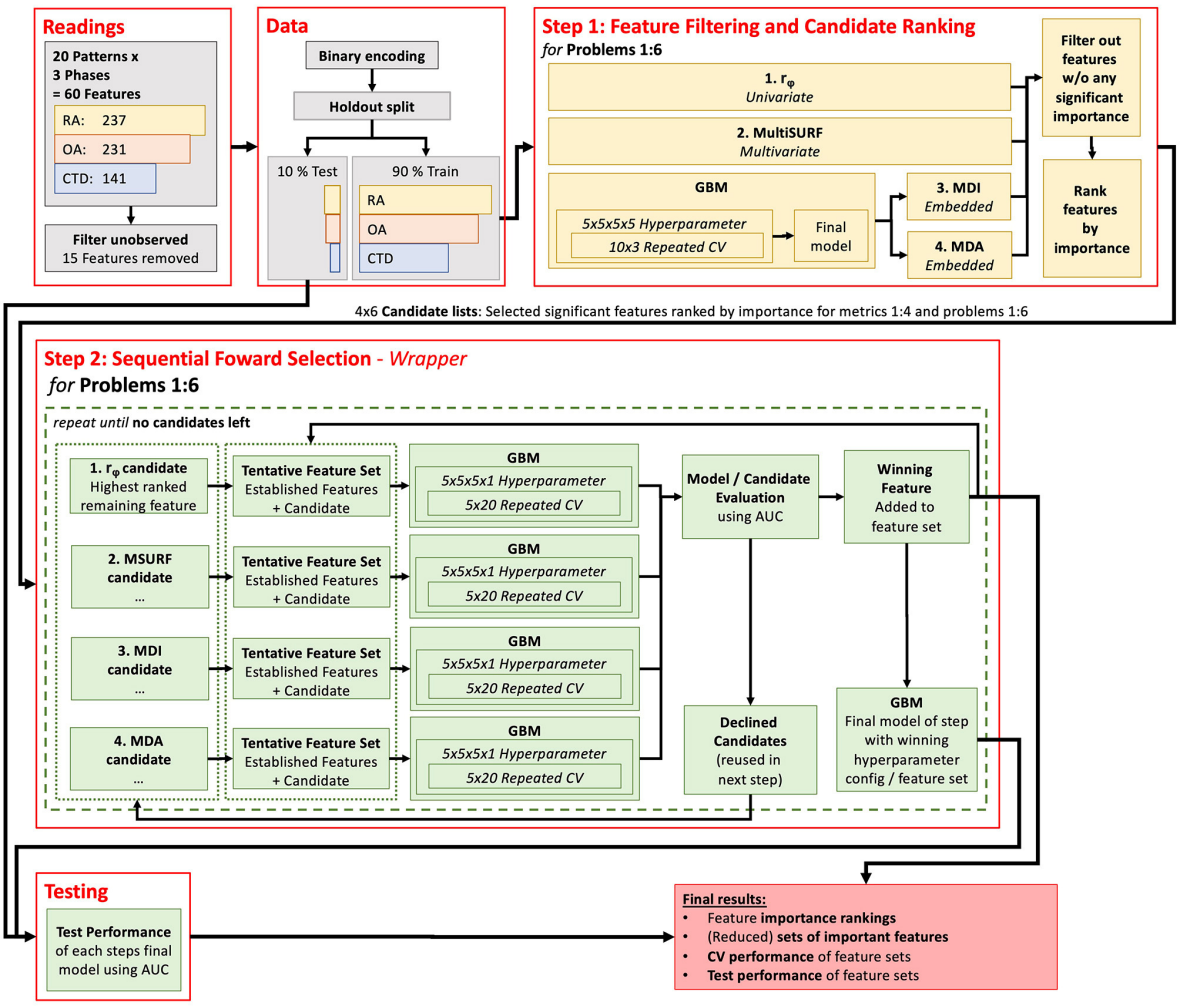
Flow diagram of the computer methods used in the presented analysis.

Assembling the right feature selection methodology can be a challenging task, as there is no one-size-fits-all approach. Instead, the applied methods need to be chosen with regard to the specific objectives of the task (14). The feature selection approach significantly reduces the feature set while ensuring that no important features are missing. Given the null hypothesis, if a feature does not provide any significant diagnostic information or, respectively, no additional diagnostic information compared to a considered feature subset, a type one error is considered less severe than a type two error. Accordingly, the removal of redundant features is desirable but not crucial, particularly because this can be harmful when no perfect correlation is present (23). Using a single feature selection method only involves the risk of missing important features due to method-specific limitations [see Pudjihartono et al. (14)]. Therefore, a two-step hybrid feature selection approach was applied using an ensemble of feature selection methods [see Pudjihartono et al. (14)]. A complete scheme of the data processing workflow is shown in Figure 1.

#### 2.5.1. Step 1: feature filtering and ranking

The first step aimed to identify features with no significant diagnostic information to remove them from further analysis and rank the features by suspected importance for a later in-depth evaluation (see 2.5.2). This reduced the search space for the final feature importance evaluation process in step 2 and sped it up.

For this purpose, two filter metrics and two embedded measures were combined: (i) the phi-coefficient, (ii) the relief algorithm MultiSURF, (iii) Mean Decrease Impurity (MDI), and (iv) Mean Decrease Accuracy (MDA).

The phi-coefficient *r*_ϕ_, which is the same as the Pearson correlation for binary variables, was used as the first filter metric. As a measure of association for two binary variables, it reveals simple univariate linear dependencies of the diagnosis on the features (and is used to examine redundancies among features as well). It is worth noting that many different measures and statistical tests are available to evaluate univariate dependencies (14). It can be shown that for all binary (dichotomous) settings, common approaches like Pearson correlation, chi-squared test, mutual information, etc., are equal or will not differ significantly in terms of feature selection in practice. Therefore, the phi-coefficient was used here as it is widely known, provides easy interpretability, and can be tested for significance using a *t*-test.

To evaluate the involvement of features in diagnostic-relevant feature interactions, a multivariate filter, namely a relief algorithm, was used. In its basic form, a relief algorithm assigns weights (*W*) to each of the input features by measuring the distance of randomly selected instances with respect to near instances of the same class and near instances of the opposite class (24). In this study, the MultiSURF algorithm is used since it is capable of detecting univariate, 2-way, and 3-way interactions (25).

Two embedded feature importance metrics were derived from a machine learning model called gradient boosting machine (GBM). A GBM consists of an ensemble of multiple single machine learning models and reaches better performance through a stage-wise combination of these (26). Decision trees are commonly used as base models (called gradient-boosted trees), as was done in this study. Decision trees recursively partition observations into sub-groups by predictor selection criteria that minimize impurity in the sub-groups and thus predict uncertainty. Thus, a regression tree's “root node” represents the best predictor using all observations, whereas subsequent nodes represent the best predictors within nested, increasingly smaller sub-samples of observations. Therefore, trees are capable of handling strong non-linear interaction effects between several predictor variables (see 2.6 for more details on the applied machine learning procedure and GBM). It is worth noting that, in contrast to step 2, all features were added to the model in this step.

The importance of a feature for the trained GBM model was investigated using the common approaches MDI and MDA. MDI is the average reduction in impurity *I*_*I*_, a feature caused by all single trees (26). MDA randomly permutes the observations of the investigated feature multiple times while leaving the other features untouched and averages the observed drop in the model's performance *I*_*A*_ (27).

These four importance estimates were subsequently used to build four reduced feature importance rankings from high to low estimated importance for all six problems containing all features that met the following criteria: *r*_ϕ_ of the feature suggested a significant association with the diagnosis (*p*-value < 0.05) or *W, I*_*A*_, or *I*_*I*_ was larger than 0. That is, the respective metric suggested a slight importance for this feature, at least. Because all features included in these lists act as candidates in the next final feature selection step, these lists are called candidate lists.

#### 2.5.2. Step 2: sequential forward selection

The second step of the feature selection process aimed to find a small, comparable set of features for each problem by which (near) maximum diagnostic performance can be reached. Therefore, a stepwise forward selection process [wrapper method, see Pudjihartono et al. (14)] was applied for each problem.

Stepwise forward feature selection was employed as a sequential machine learning procedure, commencing with a model devoid of any variables. Each step of this iterative process examines features for potential inclusion in the model. In this study, a maximum of four candidates were considered for each step. These candidates consisted of the best-ranked remaining feature from each metrics candidate list for this problem from step one. Separate GBMs were trained on a feature set consisting of a candidate feature and the yet-added features. The candidate feature that yielded the highest increase in the model's performance was finally added to the model. Importantly, candidate features not selected in a given step were not discarded but considered again as potential candidates in the following steps. This process was continued until all candidate features had been evaluated and no further candidates not already included in the model remained. At some point, adding more features to the model should not significantly improve predictive performance anymore. Conventionally, the process is aborted at this point, but in this study, all features were included to ensure no potential performance-increasing power of a feature was missed.

This kind of methodology was applied because a brute-force feature selection approach testing all possible feature subsets was not considered suitable for this large feature space. Not all available features were tested in each step, but the results of step one act as an educated guess of which features are likely to be the next most important ones.

#### 2.5.3. Gradient boosting machine

In a preliminary evaluation, different machine learning algorithms were tested on the given data to find suitable methods for this study. GBM showed the best performance and was therefore used for further analysis. All GBMs were trained and validated in this study, as described below.

The performance of a machine learning model can vary depending on the specific training and test data used. To gain insight into this performance variance, repeated cross-validation (CV), a vital technique in machine learning, was applied. During CV, multiple models are trained, and each of the training runs happens on a subset of the overall training data. The model's performance is evaluated with the remaining subset (called the validation set) of the data not used in the respective training run. The aggregated validation performance of all runs gives a more accurate estimate of the true model's performance compared to a single training and testing cycle (28). In this study, a 10 x 3 CV was used in the first step. This is a set of 30 models trained on different sampled training and validation data sets. In the second step, an even more extensive 5 x 20 CV was applied. This is caused by the differing purposes of the models. In the first step, the GBM was used only for feature importance guesses (MDI and MDA), while the exact model's performance was less interesting. In the second step of final feature ranking and selection, statistically robust and generalized findings about the model's performance were particularly crucial, especially considering that the performance differences among features can be remarkably close (see results section).

It is worth noting that hyperparameter tuning was performed during the cross-validation process to boost the GBM's classification performance. That is, model parameters that cannot be learned by themselves from the data were optimized using a grid search (29) by repeating the CV for different settings of hyperparameters. From the hyperparameter tuning, the setting with the best mean performance was chosen as the optimal set of hyperparameters (see below for details about performance measurement). Afterward, a so-called final model was trained with these optimal hyperparameters and the full set of all training data (noted as the final model in Figure 1). Finally, the performance of the models was tested on the test split set aside in advance.

Because of the unbalanced problem-dependent class distribution, a random oversampling was performed for the training set to avoid a biased classification of the models toward the majority class. That is, randomly selected instances of the minority class were copied so a balanced distribution was reached for each considered problem (30). As usual, no sampling was conducted for validation and testing.

For the performance evaluation of the GBM classifiers, the area under the receiver operating characteristics curve (ROC) was used, which is simply known as the area under the curve (AUC). The AUC was used because, unlike other common classifier performance metrics, it is independent of a specific decision threshold. The ROC is a graphical plot showing the sensitivity against “1—specificity” reached by a binary classifier when varying the decision probability threshold from 0 to 1. Therefore, the AUC can be interpreted as the probability that the classifier can distinguish between a randomly selected positive and a randomly selected negative instance (31). It is also worth noting that even though classification performance is also referred to as accuracy in this study, performance is always measured in terms of AUC, not the correct classification rate, which is often termed accuracy (ACC).

While gradient-boosted trees and other tree-based models can handle multicollinearity, correlated features can still impact the model's build and splitting processes and complicate feature importance analysis [e.g. (32–34)]. Moreover, removing highly correlated features can help reduce the dimensionality of the data set, which can help reduce overfitting, improve model interpretability, and decrease training time. Therefore, a collinearity analysis was applied in step 1.

## 3. Results

In total, data from 609 patients in three cohorts were included in the analysis. Details are listed in Table 2. Written informed consent was obtained from each patient prior to inclusion. No features were selected in the reading for four patients (2 RA, 2 OA). These patients were excluded from further analysis.

**Table 2:** Demographic data of the included cohorts.

***n =* 609**	***n***	**Sex female/male/unknown**	**Age (standard deviation)**
RA	237	179/56/2	61.7 (12.4)
OA	231	195/35/1	61.6 (10.7)
CTD	141	116/25/0	51.9 (12.5)

Features are named with a combination of a letter and a number, representing the acronym and the phase, respectively. 15 of the available features were never observed in the readings and were neglected for further analysis: E1, H1, H2, H3, O1, r2, r3, S2, S3, T1, T2, T3, W1, W2, and W3. The features B1 and U1 were never present for the diagnosis of RA and CTD and were therefore not considered for the RA-vs-CTD problem.

### 3.1. Collinearity analysis

**Figure 2:**
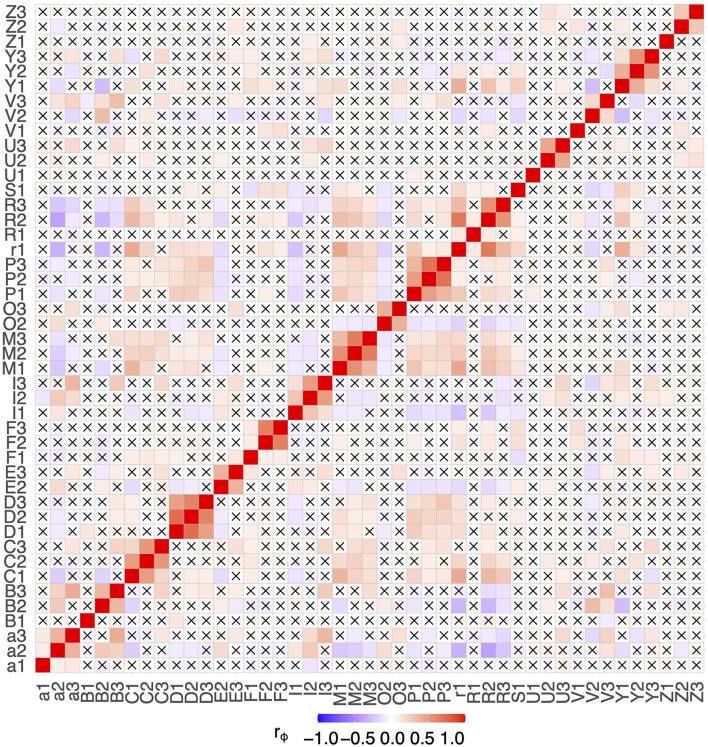
Correlation matrix of all observed features. Combinations with a *p*-value > 0.05 are marked with a cross.

The pairwise correlation matrix of the phi-coefficients for all features in all data is shown in Figure 2. No very high correlations are present among the features, which shows that no feature was completely redundant to another one in terms of contained information. Nonetheless, some features were moderately correlated among their phases, yielding potential information redundancy between these phases, e.g., D1 & D2 (*r*_ϕ_ = 0.73) and P2 & P3 (*r*_ϕ_ = 0.70). Thus, it is likely that only a little additional information was provided by using both features for a classifier. Variance inflation factor (VIF) analysis also revealed no alarming collinearities (the maximum was D2 with 3.46). Therefore, no variables were excluded from further analysis for collinearity reasons.

### 3.2. Step 1: feature filtering and ranking

**Figure 3:**
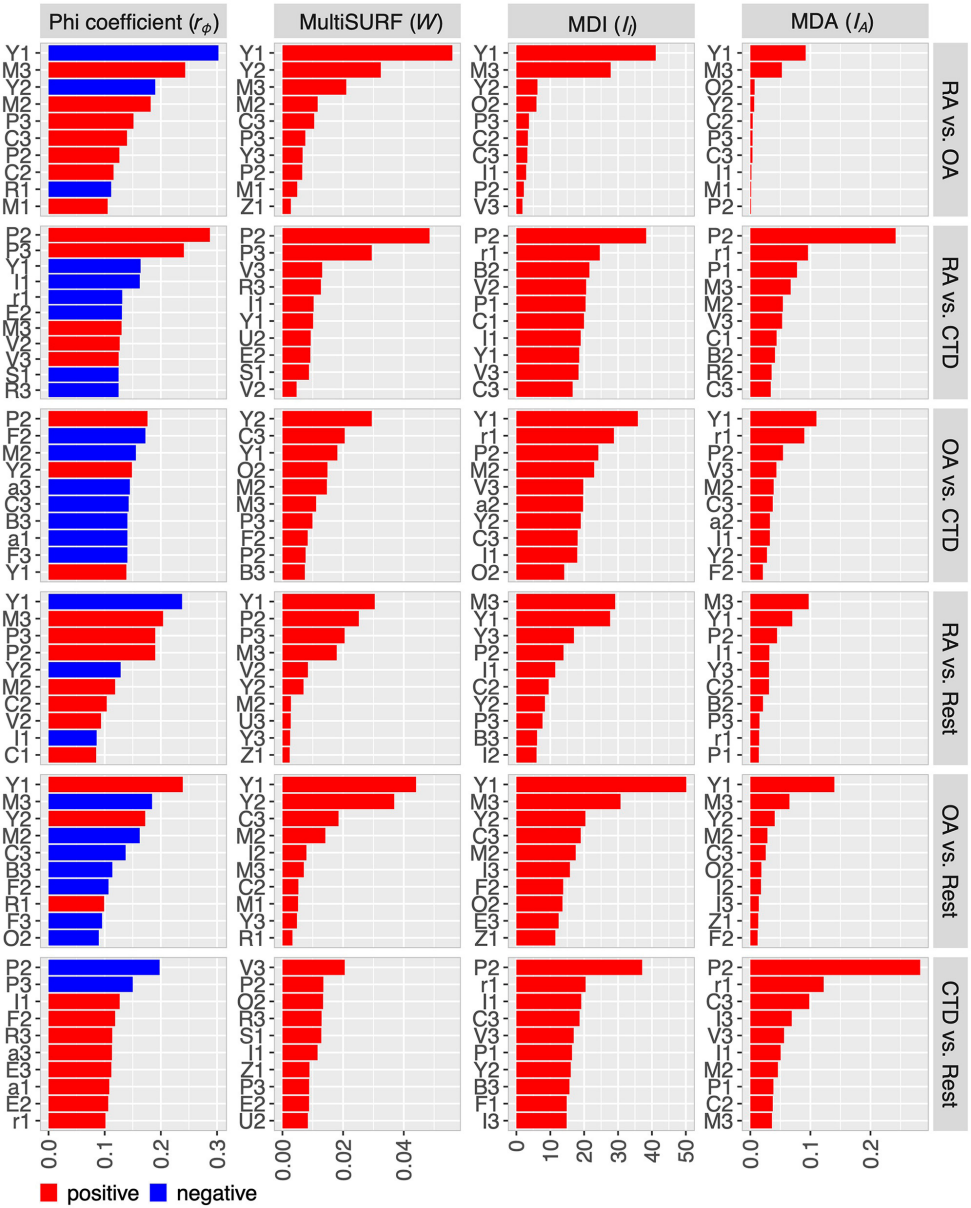
Feature ranking for the six problems with respect to the metrics phi-coefficient, MultiSURF (MSURF), mean-decrease-impurity (MDI), and mean-decrease-accuracy or permutation importance (MDA). Lists of all features are in the Supplementary material.

The four feature importance metrics of step 1 are shown in Figure 3 in descending order for the six problems. For convenience, the lists are shortened to the first 10 features. The complete lists are available in Supplementary Tables 1– 6 and visualized for each metric in Supplementary Figures 1– 6.

The results show partly noticeable differences among the used metrics regarding the ranking of the features and the number of features revealed as important, which justifies the decision to use multiple different metrics in the analysis. For RA-vs-OA, the four metrics indicated 13–18 features with at least measurable importance, reducing the number of potentially relevant features that are taken into account in step 2. For the other problems, the number of at least somehow important features is higher, depending on the respective metric. Nevertheless, reducing the feature candidates before step 2 was also feasible. Furthermore, only a few features are indicated to be of high importance, while most features only contribute to the diagnosis on a comparable low level, especially for the RA-vs-OA problem.

### 3.3. Step 2: sequential forward selection

**Figure 4:**
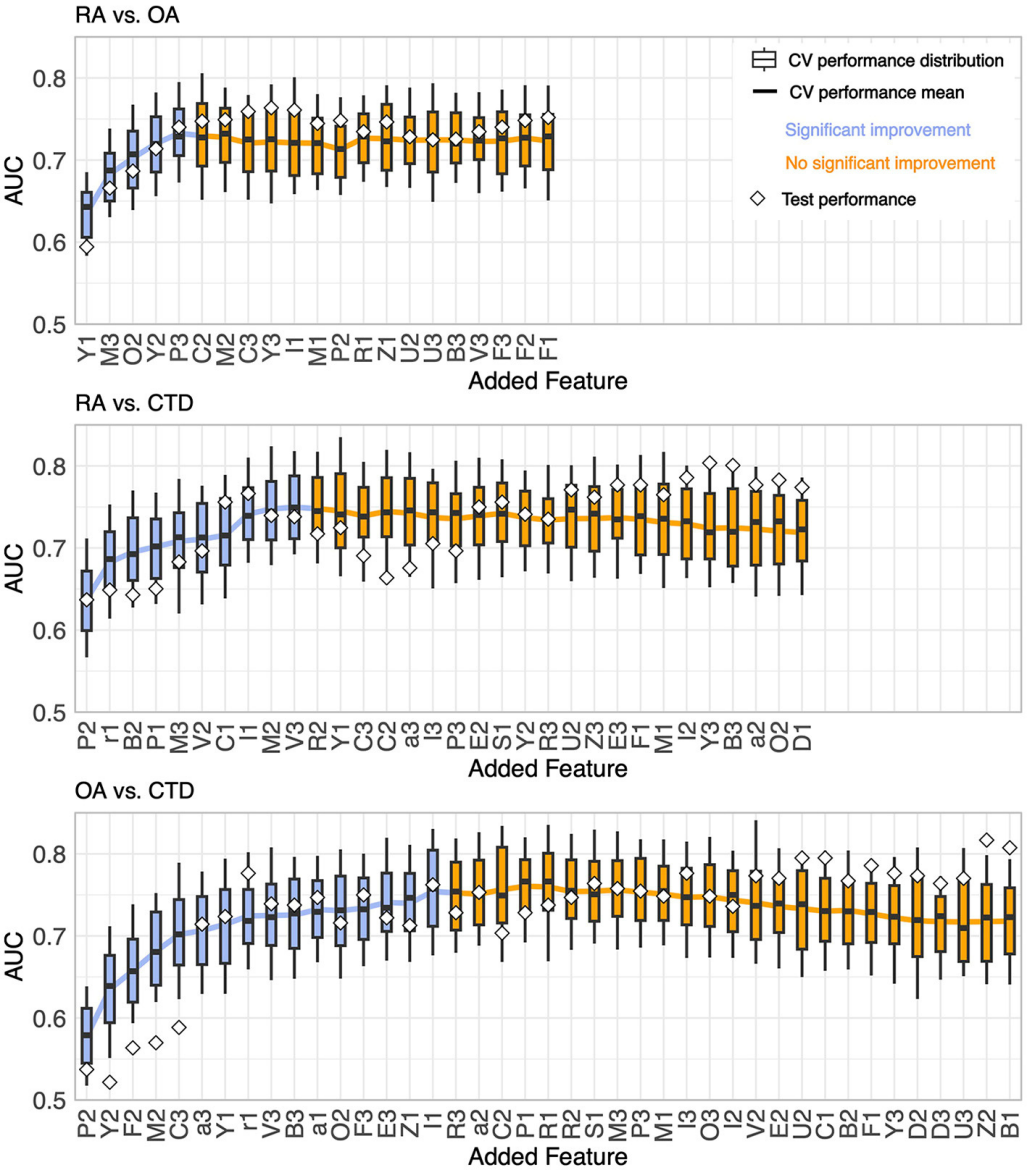
Results of the stepwise feature selection for One-vs-One problems.

**Figure 5:**
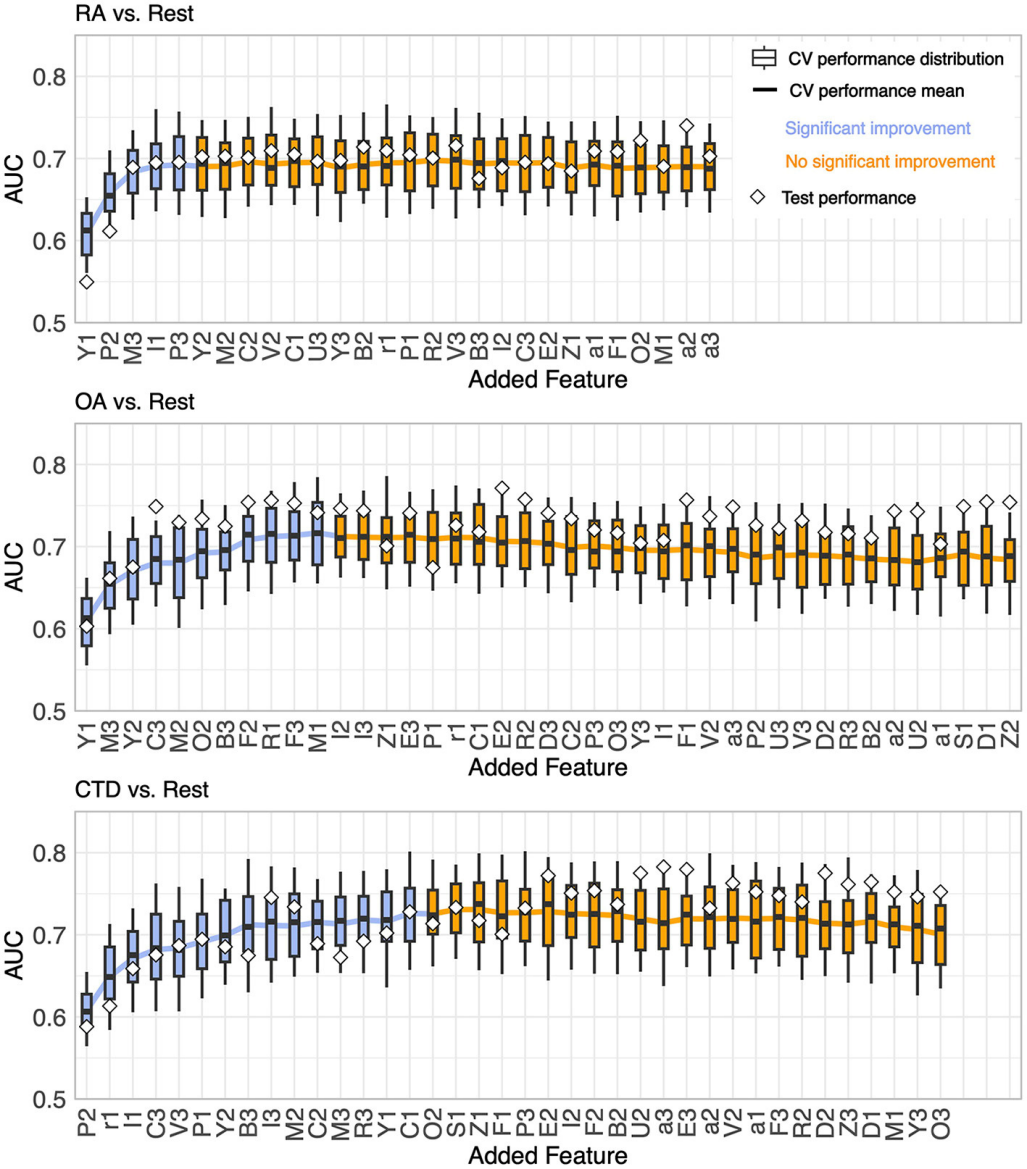
Results of the stepwise feature selection for One-vs-Rest problems.

From the resulting feature importance rankings shown in Figure 3, one feature was chosen to be most important by sequential forward selection at each step. The performances of the GBMs for the six problems are shown in Figure 4 (OvO) and Figure 5 (OvR). Each winner of a step of the selection process is presented from left to right, starting with one feature-only model on the left and adding one feature per step. This is, only the winning candidate feature newly added to the model, and the corresponding model's performance is presented. For example, for considering the OA-vs-CTD problem, the feature candidates in the first round would be P2 (suggested by the phi-coefficient ranking), Y2 (MultiSURF), and Y1 (MDI and MDA). P2 was the feature that yielded the best-performing model and therefore won the first step and was added, while all other candidates were considered in the next round again together with the next phi-coefficient feature in line (F2 in this example). The model won the second step using P2 (the winner of the previous round) and Y2 additionally (one of the new candidates tested this round), which is why only Y2 is named in the figure for this step. The boxes show the CV performance distribution of the models together with the performance of the final model of the step. Notably, the CV performance is the more reliable model's performance estimate due to its statistical robustness compared to the test performance, which relies only on a one-time random split.

For all problems, the AUC showed a rather steep rise initially as more features were incorporated, achieving a peak at a specific count and then exhibiting a mild decrease or fluctuations around the maximum with the continued addition of features. The transition point where performance ceases to show significant enhancement is marked by a shift from blue to orange in the plots. This point is defined as follows: no subsequent model demonstrates higher mean CV performance (with a tolerance range of 0.01), and the immediate succeeding model does not provide better performance either. It is worth noting that this criterion is only a suggestion by the authors and can be adjusted, as discussed in Section 4.

The number of features needed to reach the best performance was five for RA-vs-OA, ten for RA-vs-CTD, 16 for OA-vs-CTD, five for RA-vs-Rest, 11 for OA-vs-Rest, and 15 for CTD-vs-Rest, respectively. The final feature importance order, as depicted in the x-labels in Figures 4, 5, is summarized in Table 3. Features that are not included for a problem did not show sufficient performance increases. This shows that maximum diagnostic performance can be reached with a significantly reduced number of features for all problems. For all problems, the maximum performance settled around an AUC > 0.7. The test performances did not show great deviations from CV performances.

**Table 3:** Features importance lists for the six problems in descending order from left to right for each problem until no improvement considered significant is observed.

**Problem**	**Feature importance list**
RA vs. OA	Y1 M3 O2 Y2 P3
RA vs. CTD	P2 r1 B2 P1 M3 V2 C1 I1 M2 V3
OA vs. CTD	P2 Y2 F2 M2 C3 a3 Y1 r1 V3 B3 a1 O2 F3 E3 Z1 I1
RA vs. Rest	Y1 P2 M3 I1 P3
OA vs. Rest	Y1 M3 Y2 C3 M2 O2 B3 F2 R1 F3 M1
CTD vs. Rest	P2 r1 I1 C3 V3 P1 Y2 B3 I3 M2 C2 M3 R3 Y1 C1

## 4. Discussion

The presented analysis shows that feature reading of FOI is a valuable method in the differential diagnosis process for the three different rheumatic diseases RA, OA, and CTD, with a total AUC of > 0.7, which is in the range of acceptable discrimination in general (35). Of course, a good AUC depends on the specific use case, but this shows that FOI features can provide relevant diagnostic information. Thus, feature reading can be very helpful for the physician, especially in the early arthritis clinic, where one of the three diseases often appears. Moreover, the test results with the data set aside (holdout) are widely in agreement with the CV results and suggest that the learned models work for unknown data, and the presented findings generalize well.

The results reveal that only a reduced subset of known features needs to be considered to reach maximum diagnostic performance. For example, discrimination between RA and OA can already be accomplished with only five features. For RA-vs-OA, RA-vs-Rest, and OA-vs-Rest, all metrics show that only a few features are relevant and thus needed for the right diagnosis. For problems containing CTD, comparatively more features were needed to reach maximum accuracy. One reason might be that the diagnosis of CTD consists of different types of diseases, including systemic lupus erythematosus, systemic sclerosis, and others, which may be characterized by different FOI features, and thus complicate the diagnostic process, which reflects the complex nature of CTD. Nevertheless, an extensive reduction of features was also possible for CTD problems.

However, one or two features alone are not enough to reach maximum diagnostic performance. At least, the interactions of a number of features need to be considered. This is supported by the phi-coefficients from step 1, where no high or moderately high correlations for any of the problems were found (0.3 > ***r***_**ϕ**_ > −0.3), suggesting that no feature provides sufficient diagnostic information on its own. Due to the greedy one-feature-at-a-time nature of the stepwise selection algorithm, important feature interactions may be added to the models in a delayed way, making it hard to pick out these interactions accurately. An extended examination of these interactions could further reduce the number of features needed for maximum diagnostic performance.

The feature lists give no stringent instructions on how many features should be used for diagnosis, but the gain in accuracy for each additional feature hints at how much information would be added or lost. For instance, 16 features are needed for OA-vs-CTD to reach their maximum. However, after the fifth feature, C3, the performance improvement flattens, and one could decide to stop here. Moreover, in the RA-vs-Rest scenario, such flattening can be observed after the fourth feature, I1, is incorporated. It is noticeable that a total of five features are needed to achieve maximum performance. Thus, for practical FOI diagnostics, a further reduction of the suggested feature set at the price of only low performance degradation may be convenient.

Some features are found to be relevant to several problems. The most important are features P, M, Y, C, I, and B, representing the proximal interphalangeal joints (P), the metacarpophalangeal joints (M), the muscle-tendon junction of the wrist (Y), the intercarpal joints (C), an inhomogeneous signal in the nail bed (I), and broad, pronounced signals in the area of the dorsal tendons (B). They are relevant to five of the six problems. Moreover, V and F, representing superficial venous structures (V) and punctual sharp signals (F), are found five times. Interestingly, feature D, which is the distal interphalangeal joint, is not used at all in the machine algorithms, even though in the literature, increasing signal intensity at the distal joints is used to diagnose OA (4).

The expert assessment of the features found by the machine learning algorithm in the various disease groups showed high compliance with expectations from a pathological point of view, but some results deviate from experience. In the comparison of the OA-vs-CTD patient cohort, feature M, which represents an increase in fluorescence intensity in the metacarpophalangeal joints (MCP), should not have significance. Usually, they are only slightly affected in patients with OA (4), while in patients with CTD (excluding RA patients), the MCP joints are expected to be less often inflamed than the connective tissue and tendons surrounding the joints. When comparing the RA with the OA patient group, feature Y (enhancement pattern at the muscle-tendon junction of the forearm) is not expected to be remarkably important. However, the greater accumulation in the OA group could have arisen because of degenerative changes in the heavily stressed forearm in the context of OA, which would thus be consistent with the diagnosis. Furthermore, feature R, which is caused by changes in the blood flow in the fingers (Raynaud's syndrome), would have been expected to be more frequent in the CTD patient group. Some of these discrepancies can be explained by moderate correlations between features, as shown in Figure 2.

In addition, the study could not consider that two diagnoses could apply to one patient, as they were assigned to cohorts by primary diagnosis. Nevertheless, some patients have both OA and CTD or RA. Overlapping syndromes and the high variability of the pathology could have made the exact diagnosis and classification into the appropriate cohort difficult. This contradicts the exclusive classification setting of the presented problems.

A further limitation of the study is the limitation to three rheumatic diagnoses, as there are more rheumatic diseases associated with hand pain, including psoriasis arthritis, peripheral spondyloarthritis, and others. However, it could be shown that for the chosen diagnoses, the methodology provides valuable support to the diagnostic process. Another limitation is the choice of the features available in the reading, which included all known features at the time, but new findings might enlarge the set of features. Further, the readings can be extended to multiple readers to corroborate the findings.

The used statistical metrics merely expose that a feature includes high or low information for solving the problem but do not reveal if the presence or absence of the feature leads to the classification decision. In a clinical setting, this must be added by the physician. The sign of the phi-coefficient provides a hint of which feature should be present for which disease, but focusing on single features is not sufficient, as discussed above. Therefore, in future research, the specific feature interactions affecting the classification of diseases need to be addressed.

An experienced physician might miss a feature that is expected to be necessary among the ones that demonstrate the most importance. This may be because several features are correlated to some extent, as shown in Figure 2, and thus, little information is added when both of them are used. The GBM will focus on one of them. These feature correlations could also further reduce the set of necessary features, especially as some moderate correlations are present among phases 2 and 3, which suggests joining these two phases and should be investigated in future studies.

Since medical data of different kinds (e.g., laboratory data) was utilized successfully in rheumatologic patient classification tasks in the past (12), it should be evaluated if using this kind of additional data along with FOI image features could boost overall diagnostic performance further and therefore support FOI-based diagnostics in practice.

An extension to other cohorts, including psoriasis arthritis, and other diseases, and differentiating CTD diseases are already in progress.

## 5. Conclusion

Altogether, FOI feature reading is an accurate method in the process of differential diagnosis for three rheumatic diseases: RA, OA, and CTD. Therefore, it could be a helpful tool for the physician in the early arthritis clinic, in which one of the three diseases often appears. The study reveals that treating the features independently in a univariate analysis is not sufficient. Several features and feature interactions must be considered. It could be shown that for the presented problems, an extensive reduction of relevant features for the diagnostic process is available. The information gained by the calculations about which features to use in which phase for which problem and the feature-specific improvement of diagnostic performance provides helpful insight for the differential diagnostic process for the presented diseases.

## Data availability statement

The raw data supporting the conclusions of this article will be made available by the authors, without undue reservation.

## Ethics statement

The studies involving humans were approved by Ethikkommission, Ethikausschuss 1 am Campus Charité–Mitte. The studies were conducted in accordance with the local legislation and institutional requirements. The participants provided their written informed consent to participate in this study.

## Author contributions

FR, JB, PW, EG, and SO: study design. FR, JB, SK, and EG: statistical data analysis and machine learning. JB, PW, and DK: medical readings. RF, SK, EG, and SO: results review. FR, JB, PW, RF, SK, DK, EG, and SO: revision of the manuscript. All authors contributed to the article and approved the submitted version.
