# Supplementary material for: Fluorescence optical imaging feature selection with machine learning for differential diagnosis of selected rheumatic diseases
Source: Front Med (Lausanne). 2023 Aug 21;10:1228833. doi: 10.3389/fmed.2023.1228833 (PMC10475553; doi:10.3389/fmed.2023.1228833)
Supplement: Supplementary file 1 [file Data_Sheet_1.docx]

Supplementary Material

**Supplementary Figure Captions**

**Supplementary Figure 1.** Feature ranking and importance for all features for RA-vs-OA.

**Supplementary Figure 2.** Feature ranking and importance for all features for RA-vs-CTD.

**Supplementary Figure 3.** Feature ranking and importance for all features for OA-vs-CTD.

**Supplementary Figure 4.** Feature ranking and importance for all features for RA-vs-Rest.

**Supplementary Figure 5.** Feature ranking and importance for all features for OA-vs-Rest.

**Supplementary Figure 6.** Feature ranking and importance for all features for CTD-vs-Rest.
