# Supplementary figures and images for: Fluorescence optical imaging feature selection with machine learning for differential diagnosis of selected rheumatic diseases
Source: Front Med (Lausanne). 2023 Aug 21;10:1228833. doi: 10.3389/fmed.2023.1228833 (PMC10475553; doi:10.3389/fmed.2023.1228833)

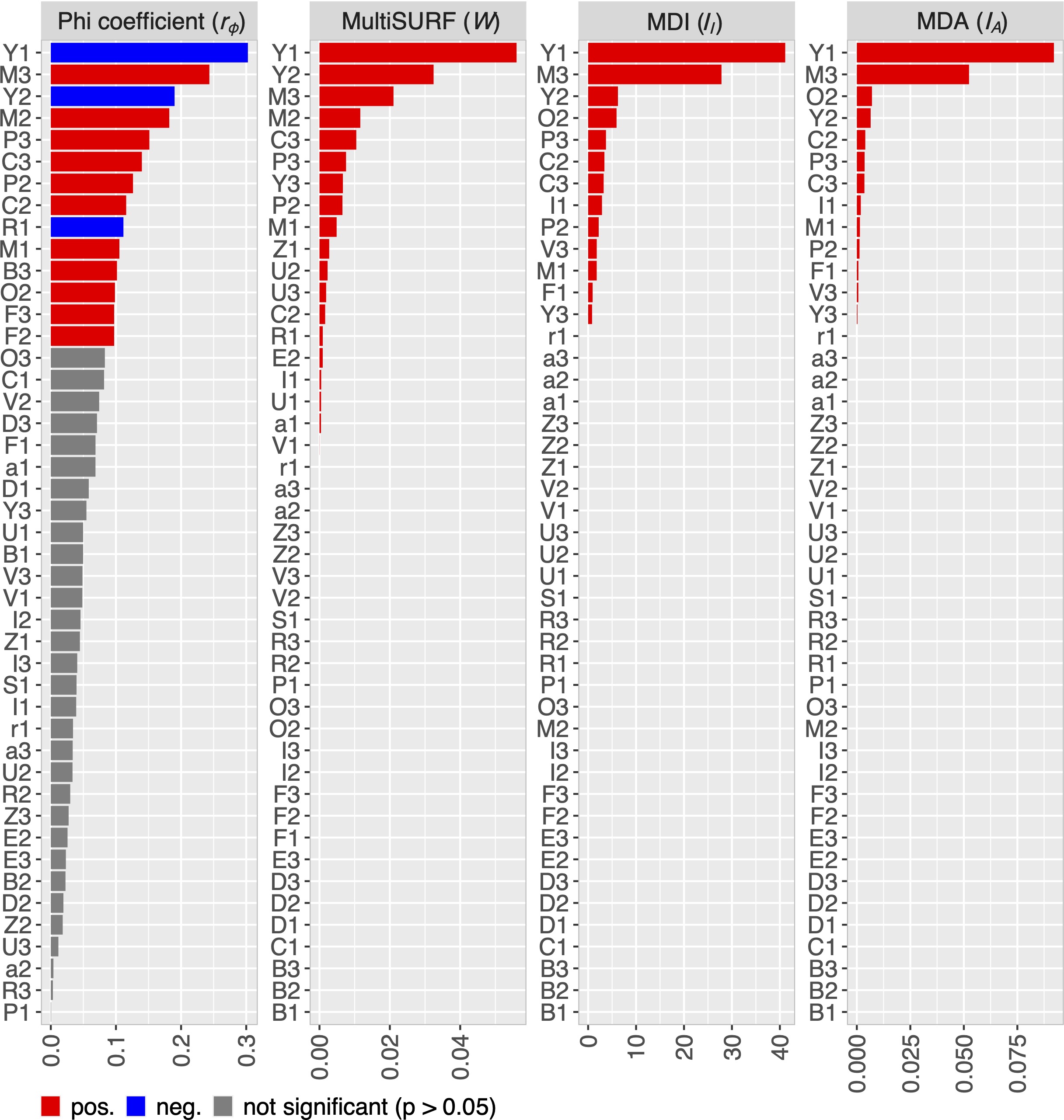

Supplement: Supplementary file 2 [file Image_1.JPEG]
