# Supplementary material for: Fluorescence optical imaging feature selection with machine learning for differential diagnosis of selected rheumatic diseases
Source: Front Med (Lausanne). 2023 Aug 21;10:1228833. doi: 10.3389/fmed.2023.1228833 (PMC10475553; doi:10.3389/fmed.2023.1228833)
Supplement: Supplementary file 8 [file Table_1.docx]

**Supplementary Table 1.** RA-vs-OA: feature importance values and ranks.

| **F** | ***r_φ_*** | ***r_φ_* p-value** | **# *r_φ_*** | ***W*** | **# *W*** | ***I_I_*** | **# *I_I_*** | ***I_A_*** | **# *I_A_*** |
| --- | --- | --- | --- | --- | --- | --- | --- | --- | --- |
| a1 | 0.068594 | 0.162064 | 20 | 0.000431 | 18 | 0 | 14 | 0 | 14 |
| a2 | -0.00401 | 0.934853 | 43 | -0.00189 | 20 | 0 | 14 | 0 | 14 |
| a3 | 0.033617 | 0.493587 | 33 | -0.01094 | 20 | 0 | 14 | 0 | 14 |
| B1 | -0.04962 | 0.312083 | 23 | -0.00069 | 20 | 0 | 14 | 0 | 14 |
| B2 | 0.022538 | 0.646293 | 39 | -0.01128 | 20 | 0 | 14 | 0 | 14 |
| B3 | 0.101575 | 0.038139 | 11 | -0.00643 | 20 | 0 | 14 | 0 | 14 |
| C1 | 0.081775 | 0.095379 | 16 | -0.00635 | 20 | 0 | 14 | 0 | 14 |
| C2 | 0.115716 | 0.018087 | 8 | 0.001618 | 13 | 3.393895 | 6 | 0.003959 | 5 |
| C3 | 0.139803 | 0.004232 | 6 | 0.010461 | 5 | 3.236904 | 7 | 0.003554 | 7 |
| D1 | -0.05828 | 0.235032 | 21 | -0.002 | 20 | 0 | 14 | 0 | 14 |
| D2 | -0.01935 | 0.693532 | 40 | -0.00472 | 20 | 0 | 14 | 0 | 14 |
| D3 | 0.070913 | 0.148302 | 18 | -0.00281 | 20 | 0 | 14 | 0 | 14 |
| E2 | -0.02572 | 0.60053 | 37 | 0.00092 | 15 | 0 | 14 | 0 | 14 |
| E3 | 0.023137 | 0.637562 | 38 | -0.00316 | 20 | 0 | 14 | 0 | 14 |
| F1 | -0.06864 | 0.161761 | 19 | -0.00099 | 20 | 0.936299 | 12 | 0.000735 | 11 |
| F2 | 0.097241 | 0.047205 | 13 | -0.0009 | 20 | 0 | 14 | 0 | 14 |
| F3 | 0.097241 | 0.047205 | 13 | -0.00107 | 20 | 0 | 14 | 0 | 14 |
| I1 | -0.039 | 0.427068 | 31 | 0.000485 | 16 | 2.887754 | 8 | 0.001855 | 8 |
| I2 | -0.0456 | 0.353004 | 27 | -0.00292 | 20 | 0 | 14 | 0 | 14 |
| I3 | -0.04058 | 0.40847 | 29 | -0.00503 | 20 | 0 | 14 | 0 | 14 |
| M1 | 0.105294 | 0.03158 | 10 | 0.004875 | 9 | 1.788391 | 11 | 0.001446 | 9 |
| M2 | 0.181878 | 0.000188 | 4 | 0.011599 | 4 | 0 | 14 | 0 | 14 |
| M3 | 0.243413 | 4.86E-07 | 2 | 0.02104 | 3 | 27.82172 | 2 | 0.052428 | 2 |
| O2 | 0.098453 | 0.044503 | 12 | -0.0011 | 20 | 5.926457 | 4 | 0.007073 | 3 |
| O3 | 0.082995 | 0.090526 | 15 | -0.00253 | 20 | 0 | 14 | 0 | 14 |
| P1 | 0.000629 | 0.989784 | 45 | -0.00418 | 20 | 0 | 14 | 0 | 14 |
| P2 | 0.126184 | 0.009899 | 7 | 0.006523 | 8 | 2.202107 | 9 | 0.001267 | 10 |
| P3 | 0.1513 | 0.001947 | 5 | 0.007551 | 6 | 3.722876 | 5 | 0.003638 | 6 |
| r1 | -0.03409 | 0.487544 | 32 | -0.00519 | 20 | 0 | 14 | 0 | 14 |
| R1 | -0.11149 | 0.022786 | 9 | 0.000933 | 14 | 0 | 14 | 0 | 14 |
| R2 | -0.02982 | 0.543658 | 35 | -0.00577 | 20 | 0 | 14 | 0 | 14 |
| R3 | -0.00322 | 0.947777 | 44 | -0.00513 | 20 | 0 | 14 | 0 | 14 |
| S1 | -0.0395 | 0.421138 | 30 | -0.00311 | 20 | 0 | 14 | 0 | 14 |
| U1 | -0.04962 | 0.312083 | 23 | 0.000453 | 17 | 0 | 14 | 0 | 14 |
| U2 | -0.03334 | 0.497223 | 34 | 0.002299 | 11 | 0 | 14 | 0 | 14 |
| U3 | 0.011649 | 0.812528 | 42 | 0.00189 | 12 | 0 | 14 | 0 | 14 |
| V1 | 0.048445 | 0.323703 | 26 | 3.32E-05 | 19 | 0 | 14 | 0 | 14 |
| V2 | 0.074262 | 0.130025 | 17 | -0.0053 | 20 | 0 | 14 | 0 | 14 |
| V3 | 0.048638 | 0.321773 | 25 | -0.008 | 20 | 1.79518 | 10 | 0.000707 | 12 |
| Y1 | -0.30255 | 2.82E-10 | 1 | 0.056022 | 1 | 41.12276 | 1 | 0.092088 | 1 |
| Y2 | -0.19 | 9.46E-05 | 3 | 0.032433 | 2 | 6.21574 | 3 | 0.006459 | 4 |
| Y3 | -0.05467 | 0.265331 | 22 | 0.006648 | 7 | 0.82075 | 13 | 0.000305 | 13 |
| Z1 | 0.044656 | 0.363024 | 28 | 0.002765 | 10 | 0 | 14 | 0 | 14 |
| Z2 | -0.01828 | 0.709675 | 41 | -0.00171 | 20 | 0 | 14 | 0 | 14 |
| Z3 | 0.027356 | 0.577485 | 36 | -0.00092 | 20 | 0 | 14 | 0 | 14 |
