# Supplementary material for: Fluorescence optical imaging feature selection with machine learning for differential diagnosis of selected rheumatic diseases
Source: Front Med (Lausanne). 2023 Aug 21;10:1228833. doi: 10.3389/fmed.2023.1228833 (PMC10475553; doi:10.3389/fmed.2023.1228833)
Supplement: Supplementary file 9 [file Table_2.docx]

**Supplementary Table 2**. RA-vs-CTD: feature importance values and ranks.

| **F** | ***r_φ_*** | ***r_φ_* p-value** | **# *r_φ_*** | ***W*** | **# *W*** | ***I_I_*** | **# *I_I_*** | ***I_A_*** | **# *I_A_*** |
| --- | --- | --- | --- | --- | --- | --- | --- | --- | --- |
| a1 | -0.08075 | 0.138456 | 20 | 0.000545 | 15 | 0 | 30 | 0 | 30 |
| a2 | -0.07273 | 0.182209 | 21 | -0.00963 | 20 | 9.587494 | 22 | 0.004755 | 23 |
| a3 | -0.11153 | 0.040435 | 13 | -0.00305 | 20 | 14.12269 | 13 | 0.016611 | 18 |
| B2 | -0.02907 | 0.594265 | 34 | -0.00922 | 20 | 21.55687 | 3 | 0.041089 | 8 |
| B3 | -0.0413 | 0.449194 | 27 | -0.0048 | 20 | 11.63563 | 18 | 0.007273 | 21 |
| C1 | 0.088553 | 0.104123 | 15 | -0.00636 | 20 | 19.96365 | 6 | 0.043741 | 7 |
| C2 | 0.085964 | 0.114681 | 16 | -0.00408 | 20 | 12.78333 | 15 | 0.030454 | 12 |
| C3 | -0.00611 | 0.910907 | 42 | -0.01305 | 20 | 16.59801 | 10 | 0.033989 | 10 |
| D1 | -0.03294 | 0.546144 | 32 | -0.00241 | 20 | 0.423556 | 29 | 0.000501 | 28 |
| D2 | 0.036052 | 0.508887 | 30 | -0.00661 | 20 | 0 | 30 | 0 | 30 |
| D3 | 0.063485 | 0.244423 | 23 | -0.00349 | 20 | 0 | 30 | 0 | 30 |
| E2 | -0.13076 | 0.01615 | 6 | 0.009189 | 8 | 8.914173 | 24 | 0.008577 | 20 |
| E3 | -0.11065 | 0.042049 | 14 | 0.000775 | 14 | 0.957887 | 28 | 0.001413 | 27 |
| F1 | -0.05883 | 0.280798 | 24 | -0.00613 | 20 | 12.25239 | 16 | 0.008933 | 19 |
| F2 | -0.08085 | 0.137978 | 19 | -0.00155 | 20 | 0 | 30 | 0 | 30 |
| F3 | -0.03995 | 0.464165 | 29 | 0.00026 | 17 | 0 | 30 | 0 | 30 |
| I1 | -0.16253 | 0.002727 | 4 | 0.010267 | 5 | 18.98968 | 7 | 0.031397 | 11 |
| I2 | -0.08551 | 0.116612 | 17 | -0.01047 | 20 | 9.595527 | 21 | 0.016727 | 17 |
| I3 | -0.03012 | 0.581058 | 33 | -0.01302 | 20 | 10.63573 | 20 | 0.026531 | 13 |
| M1 | 0.040725 | 0.455508 | 28 | -0.00682 | 20 | 8.951594 | 23 | 0.01741 | 16 |
| M2 | 0.026589 | 0.626185 | 35 | -0.00502 | 20 | 8.709632 | 25 | 0.054123 | 5 |
| M3 | 0.130068 | 0.01673 | 7 | -0.00075 | 20 | 14.54405 | 12 | 0.067069 | 4 |
| O2 | 0.009668 | 0.85944 | 40 | -0.00342 | 20 | 3.886178 | 26 | 0.002778 | 26 |
| O3 | 0.000422 | 0.99383 | 43 | -0.00599 | 20 | 0 | 30 | 0 | 30 |
| P1 | 0.018807 | 0.730457 | 37 | 0.003898 | 12 | 20.41867 | 5 | 0.077669 | 3 |
| P2 | 0.287408 | 7.52E-08 | 1 | 0.0485 | 1 | 38.30239 | 1 | 0.241837 | 1 |
| P3 | 0.241121 | 7.37E-06 | 2 | 0.02944 | 2 | 11.44147 | 19 | 0.004148 | 25 |
| r1 | -0.13127 | 0.01574 | 5 | 0.004369 | 11 | 24.61783 | 2 | 0.095991 | 2 |
| R1 | -0.07021 | 0.197858 | 22 | 0.000261 | 16 | 0 | 30 | 0 | 30 |
| R2 | -0.11153 | 0.040435 | 12 | -0.00391 | 20 | 15.45854 | 11 | 0.035385 | 9 |
| R3 | -0.1247 | 0.021843 | 10 | 0.012674 | 4 | 0 | 30 | 0 | 30 |
| S1 | -0.1247 | 0.021843 | 10 | 0.008741 | 9 | 1.786911 | 27 | 0.004502 | 24 |
| U2 | -0.05338 | 0.327824 | 25 | 0.009306 | 7 | 0 | 30 | 0 | 30 |
| U3 | -0.01621 | 0.766475 | 38 | 2.83E-05 | 19 | 0 | 30 | 0 | 30 |
| V1 | -0.01979 | 0.716896 | 36 | 0.000181 | 18 | 0 | 30 | 0 | 30 |
| V2 | 0.126751 | 0.01975 | 8 | 0.004664 | 10 | 20.59014 | 4 | 8.53E-05 | 29 |
| V3 | 0.124808 | 0.021731 | 9 | 0.013092 | 3 | 18.34257 | 9 | 0.052962 | 6 |
| Y1 | -0.16394 | 0.0025 | 3 | 0.010105 | 6 | 18.54685 | 8 | 0.024507 | 14 |
| Y2 | -0.03597 | 0.509829 | 31 | -0.00729 | 20 | 14.01867 | 14 | 0.019553 | 15 |
| Y3 | -0.04448 | 0.414971 | 26 | -0.00386 | 20 | 12.23892 | 17 | 0.006375 | 22 |
| Z1 | -0.08146 | 0.135039 | 18 | -0.00023 | 20 | 0 | 30 | 0 | 30 |
| Z2 | -0.01586 | 0.771366 | 39 | -0.00026 | 20 | 0 | 30 | 0 | 30 |
| Z3 | 0.008286 | 0.879365 | 41 | 0.002882 | 13 | 0 | 30 | 0 | 30 |
