# Supplementary material for: Fluorescence optical imaging feature selection with machine learning for differential diagnosis of selected rheumatic diseases
Source: Front Med (Lausanne). 2023 Aug 21;10:1228833. doi: 10.3389/fmed.2023.1228833 (PMC10475553; doi:10.3389/fmed.2023.1228833)
Supplement: Supplementary file 10 [file Table_3.docx]

**Supplementary Table 3.** OA-vs-CTD feature importance values and ranks.

| **F** | ***r_φ_*** | ***r_φ_* p-value** | **# *r_φ_*** | ***W*** | **# *W*** | ***I_I_*** | **# *I_I_*** | ***I_A_*** | **# *I_A_*** |
| --- | --- | --- | --- | --- | --- | --- | --- | --- | --- |
| a1 | -0.1404 | 0.0103 | 8 | 0.0041 | 17 | 4.9109 | 27 | 0.0070 | 21 |
| a2 | -0.0691 | 0.2086 | 25 | -0.0177 | 23 | 19.6917 | 6 | 0.0326 | 7 |
| a3 | -0.1449 | 0.0081 | 5 | 0.0048 | 15 | 13.7244 | 11 | 0.0024 | 27 |
| B1 | 0.0431 | 0.4332 | 31 | -0.0005 | 23 | 0.3316 | 41 | 0.0003 | 37 |
| B2 | -0.0511 | 0.3529 | 30 | -0.0098 | 23 | 3.0941 | 32 | 0.0006 | 33 |
| B3 | -0.1405 | 0.0103 | 7 | 0.0074 | 10 | 10.6095 | 14 | 0.0087 | 20 |
| C1 | 0.0106 | 0.8476 | 40 | -0.0077 | 23 | 3.1475 | 31 | 0.0002 | 38 |
| C2 | -0.0260 | 0.6362 | 35 | -0.0037 | 23 | 10.7687 | 13 | 0.0169 | 13 |
| C3 | -0.1427 | 0.0091 | 6 | 0.0205 | 2 | 18.0705 | 8 | 0.0373 | 6 |
| D1 | 0.0239 | 0.6643 | 36 | -0.0102 | 23 | 0.0000 | 42 | 0.0000 | 42 |
| D2 | 0.0543 | 0.3228 | 29 | -0.0077 | 23 | 1.5087 | 37 | 0.0008 | 30 |
| D3 | -0.0037 | 0.9464 | 44 | -0.0025 | 23 | 1.3501 | 38 | 0.0009 | 29 |
| E2 | -0.1052 | 0.0551 | 17 | -0.0031 | 23 | 6.4787 | 23 | 0.0006 | 35 |
| E3 | -0.1341 | 0.0143 | 11 | -0.0002 | 23 | 8.4939 | 20 | 0.0089 | 19 |
| F1 | 0.0079 | 0.8856 | 43 | -0.0088 | 23 | 2.1600 | 34 | 0.0001 | 41 |
| F2 | -0.1725 | 0.0016 | 2 | 0.0083 | 8 | 13.1968 | 12 | 0.0208 | 10 |
| F3 | -0.1404 | 0.0103 | 8 | 0.0067 | 13 | 2.6120 | 33 | 0.0046 | 23 |
| I1 | -0.1248 | 0.0228 | 13 | 0.0073 | 11 | 17.9633 | 9 | 0.0325 | 8 |
| I2 | -0.0416 | 0.4495 | 33 | 0.0024 | 19 | 4.3141 | 29 | 0.0007 | 31 |
| I3 | 0.0094 | 0.8649 | 41 | -0.0019 | 23 | 3.5286 | 30 | 0.0041 | 24 |
| M1 | -0.0621 | 0.2584 | 26 | -0.0050 | 23 | 6.4433 | 24 | 0.0069 | 22 |
| M2 | -0.1556 | 0.0044 | 3 | 0.0146 | 5 | 22.9602 | 4 | 0.0388 | 5 |
| M3 | -0.1139 | 0.0377 | 15 | 0.0111 | 6 | 7.3360 | 22 | 0.0002 | 40 |
| O2 | -0.0889 | 0.1054 | 19 | 0.0148 | 4 | 14.1099 | 10 | 0.0163 | 14 |
| O3 | -0.0838 | 0.1271 | 21 | 0.0042 | 16 | 5.2212 | 26 | 0.0041 | 25 |
| P1 | 0.0183 | 0.7399 | 39 | 0.0006 | 21 | 9.9750 | 18 | 0.0193 | 11 |
| P2 | 0.1763 | 0.0012 | 1 | 0.0076 | 9 | 24.1816 | 3 | 0.0544 | 3 |
| P3 | 0.1056 | 0.0543 | 16 | 0.0099 | 7 | 1.6537 | 36 | 0.0005 | 36 |
| r1 | -0.0980 | 0.0741 | 18 | -0.0090 | 23 | 28.7858 | 2 | 0.0899 | 2 |
| R1 | 0.0599 | 0.2759 | 27 | -0.0017 | 23 | 10.1796 | 17 | 0.0172 | 12 |
| R2 | -0.0822 | 0.1345 | 22 | -0.0093 | 23 | 10.2009 | 16 | 0.0138 | 16 |
| R3 | -0.1216 | 0.0265 | 14 | 0.0068 | 12 | 7.9266 | 21 | 0.0092 | 18 |
| S1 | -0.0850 | 0.1215 | 20 | 0.0026 | 18 | 8.5769 | 19 | 0.0095 | 17 |
| U1 | 0.0431 | 0.4332 | 31 | -0.0010 | 23 | 0.0000 | 42 | 0.0000 | 42 |
| U2 | -0.0202 | 0.7128 | 37 | -0.0012 | 23 | 4.6890 | 28 | 0.0023 | 28 |
| U3 | -0.0278 | 0.6127 | 34 | -0.0009 | 23 | 1.0390 | 39 | 0.0007 | 32 |
| V1 | -0.0699 | 0.2033 | 24 | -0.0002 | 23 | 0.0000 | 42 | 0.0000 | 42 |
| V2 | 0.0553 | 0.3144 | 28 | -0.0199 | 23 | 6.3473 | 25 | 0.0033 | 26 |
| V3 | 0.0767 | 0.1627 | 23 | 0.0021 | 20 | 19.7364 | 5 | 0.0433 | 4 |
| Y1 | 0.1386 | 0.0113 | 10 | 0.0181 | 3 | 35.8566 | 1 | 0.1100 | 1 |
| Y2 | 0.1486 | 0.0066 | 4 | 0.0294 | 1 | 19.0089 | 7 | 0.0277 | 9 |
| Y3 | 0.0085 | 0.8766 | 42 | -0.0025 | 23 | 2.1241 | 35 | 0.0006 | 34 |
| Z1 | -0.1267 | 0.0207 | 12 | 0.0056 | 14 | 10.2122 | 15 | 0.0160 | 15 |
| Z2 | 0.0021 | 0.9701 | 45 | -0.0042 | 23 | 0.7496 | 40 | 0.0002 | 39 |
| Z3 | -0.0190 | 0.7300 | 38 | 0.0003 | 22 | 0.0000 | 42 | 0.0000 | 42 |
