# Supplementary material for: Fluorescence optical imaging feature selection with machine learning for differential diagnosis of selected rheumatic diseases
Source: Front Med (Lausanne). 2023 Aug 21;10:1228833. doi: 10.3389/fmed.2023.1228833 (PMC10475553; doi:10.3389/fmed.2023.1228833)
Supplement: Supplementary file 11 [file Table_4.docx]

**Supplementary Table 4.** RA-vs-Rest: feature importance values and ranks.

| **F** | ***r_φ_*** | ***r_φ_* p-value** | **# *r_φ_*** | ***W*** | **# *W*** | ***I_I_*** | **# *I_I_*** | ***I_A_*** | **# *I_A_*** |
| --- | --- | --- | --- | --- | --- | --- | --- | --- | --- |
| a1 | -0.01182 | 0.783281 | 39 | 0.001435 | 11 | 0 | 26 | 0 | 26 |
| a2 | -0.03025 | 0.481372 | 33 | -0.00251 | 20 | 2.535383 | 22 | 0.002783 | 23 |
| a3 | -0.02379 | 0.579761 | 35 | -0.00459 | 20 | 1.522087 | 24 | 0.002547 | 24 |
| B1 | -0.03416 | 0.42653 | 30 | -0.00073 | 20 | 0 | 26 | 0 | 26 |
| B2 | 0.002403 | 0.955405 | 43 | -0.00243 | 20 | 5.675278 | 12 | 0.021105 | 7 |
| B3 | 0.044917 | 0.295675 | 27 | -0.00081 | 20 | 6.107241 | 9 | 0.007199 | 15 |
| C1 | 0.084723 | 0.04826 | 10 | -0.00484 | 20 | 3.629887 | 18 | 0.004378 | 20 |
| C2 | 0.103621 | 0.015615 | 7 | 0.00131 | 12 | 9.511596 | 6 | 0.031081 | 6 |
| C3 | 0.081561 | 0.057288 | 12 | -0.00618 | 20 | 3.571243 | 19 | 0.005794 | 17 |
| D1 | -0.04712 | 0.27256 | 26 | -0.00193 | 20 | 0 | 26 | 0 | 26 |
| D2 | 0.001405 | 0.973918 | 44 | -0.00399 | 20 | 0 | 26 | 0 | 26 |
| D3 | 0.06966 | 0.104598 | 17 | 0.00067 | 15 | 0 | 26 | 0 | 26 |
| E2 | -0.06573 | 0.125705 | 18 | -0.00552 | 20 | 3.634072 | 17 | 0.005318 | 19 |
| E3 | -0.03253 | 0.448942 | 32 | -0.00564 | 20 | 0 | 26 | 0 | 26 |
| F1 | -0.06365 | 0.138175 | 19 | -0.00302 | 20 | 3.336346 | 20 | 0.00547 | 18 |
| F2 | 0.003407 | 0.936803 | 42 | -0.00081 | 20 | 0 | 26 | 0 | 26 |
| F3 | 0.028114 | 0.512882 | 34 | -0.00108 | 20 | 0 | 26 | 0 | 26 |
| I1 | -0.08568 | 0.045787 | 9 | 0.000622 | 16 | 11.46581 | 5 | 0.031654 | 4 |
| I2 | -0.06032 | 0.160008 | 22 | -0.00968 | 20 | 5.951022 | 10 | 0.00642 | 16 |
| I3 | -0.03579 | 0.404795 | 29 | -0.00336 | 20 | 0 | 26 | 0 | 26 |
| M1 | 0.079461 | 0.064026 | 13 | 0.000345 | 18 | 1.897274 | 23 | 0.00372 | 21 |
| M2 | 0.118545 | 0.005635 | 6 | 0.002802 | 7 | 0.508734 | 25 | 0.000749 | 25 |
| M3 | 0.203989 | 1.61E-06 | 2 | 0.01789 | 4 | 29.14026 | 1 | 0.097077 | 1 |
| O2 | 0.062066 | 0.148268 | 20 | 0.001131 | 13 | 2.922358 | 21 | 0.00294 | 22 |
| O3 | 0.048433 | 0.259441 | 25 | -0.00019 | 20 | 0 | 26 | 0 | 26 |
| P1 | 0.007568 | 0.860215 | 41 | -0.00591 | 20 | 5.356491 | 15 | 0.014166 | 10 |
| P2 | 0.189969 | 8.15E-06 | 4 | 0.025196 | 2 | 13.89972 | 4 | 0.044459 | 3 |
| P3 | 0.190007 | 8.11E-06 | 3 | 0.020492 | 3 | 7.720199 | 8 | 0.015277 | 8 |
| r1 | -0.07069 | 0.099536 | 15 | 0.000344 | 19 | 5.564927 | 14 | 0.014787 | 9 |
| R1 | -0.08406 | 0.05004 | 11 | -0.00168 | 20 | 0 | 26 | 0 | 26 |
| R2 | -0.06049 | 0.158832 | 21 | 0.000707 | 14 | 4.966204 | 16 | 0.010965 | 12 |
| R3 | -0.05209 | 0.225105 | 23 | -0.00281 | 20 | 0 | 26 | 0 | 26 |
| S1 | -0.07043 | 0.100814 | 16 | -0.0033 | 20 | 0 | 26 | 0 | 26 |
| U1 | -0.03416 | 0.42653 | 30 | -0.00016 | 20 | 0 | 26 | 0 | 26 |
| U2 | -0.0396 | 0.356616 | 28 | -0.00085 | 20 | 0 | 26 | 0 | 26 |
| U3 | 0.000388 | 0.992802 | 45 | 0.00272 | 8 | 0 | 26 | 0 | 26 |
| V1 | 0.013979 | 0.744944 | 38 | 0.000496 | 17 | 0 | 26 | 0 | 26 |
| V2 | 0.093462 | 0.029284 | 8 | 0.008393 | 5 | 5.775143 | 11 | 0.009239 | 13 |
| V3 | 0.076165 | 0.075905 | 14 | -0.00931 | 20 | 5.576677 | 13 | 0.007965 | 14 |
| Y1 | -0.23789 | 1.95E-08 | 1 | 0.030432 | 1 | 27.65349 | 2 | 0.069772 | 2 |
| Y2 | -0.12842 | 0.002692 | 5 | 0.006944 | 6 | 8.422413 | 7 | 0.012404 | 11 |
| Y3 | -0.04999 | 0.244399 | 24 | 0.002524 | 9 | 16.98781 | 3 | 0.031104 | 5 |
| Z1 | -0.01051 | 0.806755 | 40 | 0.002376 | 10 | 0 | 26 | 0 | 26 |
| Z2 | -0.01681 | 0.695668 | 37 | -0.00209 | 20 | 0 | 26 | 0 | 26 |
| Z3 | 0.019806 | 0.644844 | 36 | -0.00149 | 20 | 0 | 26 | 0 | 26 |
