# Supplementary material for: Fluorescence optical imaging feature selection with machine learning for differential diagnosis of selected rheumatic diseases
Source: Front Med (Lausanne). 2023 Aug 21;10:1228833. doi: 10.3389/fmed.2023.1228833 (PMC10475553; doi:10.3389/fmed.2023.1228833)
Supplement: Supplementary file 12 [file Table_5.docx]

**Supplementary Table 5.** OA-vs-Rest: feature importance values and ranks.

| **F** | ***r_φ_*** | ***r_φ_* p-value** | **# *r_φ_*** | ***W*** | **# *W*** | ***I_I_*** | **# *I_I_*** | ***I_A_*** | **# *I_A_*** |
| --- | --- | --- | --- | --- | --- | --- | --- | --- | --- |
| a1 | -0.08244 | 0.054636 | 12 | -0.00251 | 18 | 1.540714 | 37 | 0.001364 | 38 |
| a2 | -0.02337 | 0.586562 | 34 | 0.000229 | 16 | 3.492228 | 33 | 0.003081 | 35 |
| a3 | -0.07458 | 0.082237 | 15 | -0.00509 | 18 | 6.53785 | 18 | 0.004975 | 27 |
| B1 | 0.05497 | 0.200501 | 19 | -1.8E-05 | 18 | 0 | 42 | 0 | 42 |
| B2 | -0.03279 | 0.445296 | 28 | -0.01051 | 18 | 3.759793 | 32 | 0.003789 | 32 |
| B3 | -0.11359 | 0.008006 | 6 | -0.00103 | 18 | 1.825089 | 36 | 0.00479 | 29 |
| C1 | -0.04646 | 0.279394 | 23 | -0.00316 | 18 | 10.10505 | 13 | 0.007459 | 15 |
| C2 | -0.07988 | 0.062636 | 13 | 0.005249 | 7 | 4.304131 | 28 | 0.007417 | 16 |
| C3 | -0.13735 | 0.001321 | 5 | 0.018484 | 3 | 19.00232 | 4 | 0.025618 | 5 |
| D1 | 0.044582 | 0.29929 | 25 | -0.00167 | 18 | 1.338763 | 40 | 0.000965 | 40 |
| D2 | 0.032395 | 0.450827 | 29 | -0.00307 | 18 | 4.098247 | 30 | 0.003397 | 34 |
| D3 | -0.04532 | 0.291328 | 24 | -0.00547 | 18 | 6.893971 | 17 | 0.007087 | 18 |
| E2 | -0.02666 | 0.534979 | 31 | 0.001257 | 13 | 9.331508 | 14 | 0.007318 | 17 |
| E3 | -0.06479 | 0.131219 | 17 | -0.00204 | 18 | 12.46633 | 9 | 0.01137 | 11 |
| F1 | 0.044454 | 0.300686 | 26 | 0.00172 | 12 | 5.972113 | 22 | 0.006317 | 23 |
| F2 | -0.10683 | 0.012661 | 7 | -0.00105 | 18 | 13.81051 | 7 | 0.01216 | 10 |
| F3 | -0.09538 | 0.026116 | 9 | -0.00166 | 18 | 1.42894 | 38 | 0.001213 | 39 |
| I1 | -0.02445 | 0.569331 | 32 | 0.001865 | 11 | 3.120246 | 34 | 0.003666 | 33 |
| I2 | 0.012072 | 0.778758 | 41 | 0.007908 | 5 | 10.97732 | 11 | 0.017751 | 7 |
| I3 | 0.028074 | 0.513493 | 30 | -0.00247 | 18 | 15.79487 | 6 | 0.014138 | 8 |
| M1 | -0.08615 | 0.044584 | 11 | 0.005166 | 8 | 4.742133 | 27 | 0.005134 | 26 |
| M2 | -0.16239 | 0.000142 | 4 | 0.014133 | 4 | 17.50995 | 5 | 0.028442 | 4 |
| M3 | -0.18441 | 1.5E-05 | 2 | 0.007054 | 6 | 30.72989 | 2 | 0.065069 | 2 |
| O2 | -0.08948 | 0.036945 | 10 | -0.00746 | 18 | 13.59826 | 8 | 0.018365 | 6 |
| O3 | -0.07747 | 0.070992 | 14 | -0.00561 | 18 | 6.137597 | 21 | 0.006593 | 20 |
| P1 | 0.006432 | 0.881013 | 44 | -0.00164 | 18 | 10.47445 | 12 | 0.010487 | 12 |
| P2 | -0.0184 | 0.668566 | 36 | -0.00166 | 18 | 5.684431 | 23 | 0.006404 | 22 |
| P3 | -0.06006 | 0.161882 | 18 | 0.000227 | 17 | 4.192741 | 29 | 0.008089 | 14 |
| r1 | -0.01738 | 0.685883 | 37 | -0.00527 | 18 | 6.243756 | 20 | 0.008146 | 13 |
| R1 | 0.098986 | 0.020938 | 8 | 0.003298 | 10 | 7.137712 | 16 | 0.00546 | 25 |
| R2 | -0.01429 | 0.739493 | 39 | -0.00491 | 18 | 8.440933 | 15 | 0.006751 | 19 |
| R3 | -0.04657 | 0.278217 | 22 | -0.00324 | 18 | 3.97945 | 31 | 0.003803 | 31 |
| S1 | -0.01225 | 0.775597 | 40 | -0.00388 | 18 | 1.398512 | 39 | 0.002061 | 37 |
| U1 | 0.05497 | 0.200501 | 19 | 0.000985 | 14 | 0 | 42 | 0 | 42 |
| U2 | 0.011528 | 0.7885 | 43 | 0.000965 | 15 | 3.054098 | 35 | 0.002628 | 36 |
| U3 | -0.01729 | 0.687365 | 38 | -0.00103 | 18 | 5.078958 | 26 | 0.004883 | 28 |
| V1 | -0.04742 | 0.269521 | 21 | -0.0006 | 18 | 0 | 42 | 0 | 42 |
| V2 | -0.02437 | 0.570577 | 33 | -0.01214 | 18 | 6.262246 | 19 | 0.006553 | 21 |
| V3 | 0.001575 | 0.970768 | 45 | -0.00682 | 18 | 5.565032 | 25 | 0.004759 | 30 |
| Y1 | 0.239133 | 1.63E-08 | 1 | 0.044062 | 1 | 50.1376 | 1 | 0.139864 | 1 |
| Y2 | 0.17217 | 5.42E-05 | 3 | 0.03685 | 2 | 20.37449 | 3 | 0.040586 | 3 |
| Y3 | 0.036306 | 0.398046 | 27 | 0.004788 | 9 | 5.652083 | 24 | 0.005927 | 24 |
| Z1 | -0.07239 | 0.091652 | 16 | -0.00237 | 18 | 11.48843 | 10 | 0.013237 | 9 |
| Z2 | 0.011763 | 0.78429 | 42 | -0.00011 | 18 | 0.392534 | 41 | 0.000152 | 41 |
| Z3 | -0.02283 | 0.595164 | 35 | -0.0006 | 18 | 0 | 42 | 0 | 42 |
