# Supplementary material for: Fluorescence optical imaging feature selection with machine learning for differential diagnosis of selected rheumatic diseases
Source: Front Med (Lausanne). 2023 Aug 21;10:1228833. doi: 10.3389/fmed.2023.1228833 (PMC10475553; doi:10.3389/fmed.2023.1228833)
Supplement: Supplementary file 13 [file Table_6.docx]

**Supplementary Table 6.** CTD-vs-Rest: feature importance values and ranks.

| **F** | ***r_φ_*** | ***r_φ_* p-value** | **# *r_φ_*** | ***W*** | **# *W*** | ***I_I_*** | **# *I_I_*** | ***I_A_*** | **# *I_A_*** |
| --- | --- | --- | --- | --- | --- | --- | --- | --- | --- |
| a1 | 0.108147 | 0.011603 | 8 | 0.004932 | 14 | 0 | 32 | 0 | 32 |
| a2 | 0.061637 | 0.151096 | 19 | -0.01781 | 25 | 12.22673 | 14 | 0.010736 | 19 |
| a3 | 0.112918 | 0.008388 | 6 | 0.002707 | 19 | 12.92082 | 13 | 0.015426 | 16 |
| B1 | -0.02368 | 0.58151 | 32 | -0.00039 | 25 | 0 | 32 | 0 | 32 |
| B2 | 0.034832 | 0.417482 | 26 | -0.01023 | 25 | 13.20112 | 12 | 0.014337 | 17 |
| B3 | 0.078507 | 0.067296 | 16 | -0.00056 | 25 | 15.66331 | 8 | 0.007594 | 23 |
| C1 | -0.04432 | 0.302177 | 23 | -0.00762 | 25 | 13.36102 | 11 | 0.017757 | 14 |
| C2 | -0.02776 | 0.518144 | 31 | -0.01179 | 25 | 12.16916 | 15 | 0.037362 | 9 |
| C3 | 0.063546 | 0.138812 | 18 | 0.003717 | 17 | 18.64574 | 4 | 0.098095 | 3 |
| D1 | 0.00316 | 0.941388 | 44 | -0.00713 | 25 | 3.519159 | 29 | 0.008279 | 22 |
| D2 | -0.03876 | 0.36686 | 24 | -0.00754 | 25 | 2.887537 | 31 | 0.01331 | 18 |
| D3 | -0.02827 | 0.510574 | 30 | -0.00302 | 25 | 0 | 32 | 0 | 32 |
| E2 | 0.106279 | 0.013132 | 9 | 0.008776 | 9 | 11.57433 | 17 | 0.004967 | 25 |
| E3 | 0.111762 | 0.009083 | 7 | 0.004006 | 16 | 6.898001 | 26 | 0.008606 | 21 |
| F1 | 0.022341 | 0.603099 | 35 | -0.0073 | 25 | 14.84403 | 9 | 5.25E-05 | 30 |
| F2 | 0.118573 | 0.005623 | 4 | 0.006956 | 12 | 0 | 32 | 0 | 32 |
| F3 | 0.076977 | 0.072825 | 17 | 0.00744 | 11 | 0 | 32 | 0 | 32 |
| I1 | 0.12672 | 0.003069 | 3 | 0.011567 | 6 | 19.14871 | 3 | 0.050468 | 6 |
| I2 | 0.055642 | 0.195043 | 20 | -0.0086 | 25 | 8.57383 | 20 | 0.016702 | 15 |
| I3 | 0.009034 | 0.8335 | 40 | -0.00621 | 25 | 14.82813 | 10 | 0.0689 | 4 |
| M1 | 0.00726 | 0.86584 | 41 | -0.009 | 25 | 7.958431 | 22 | 0.00303 | 26 |
| M2 | 0.049651 | 0.247641 | 21 | 0.001698 | 22 | 11.43793 | 19 | 0.045874 | 7 |
| M3 | -0.02352 | 0.584145 | 34 | 0.002503 | 20 | 11.4992 | 18 | 0.035992 | 10 |
| O2 | 0.031108 | 0.469021 | 29 | 0.013391 | 3 | 5.409511 | 28 | 0.001517 | 27 |
| O3 | 0.033045 | 0.441787 | 27 | 0.002005 | 21 | 0 | 32 | 0 | 32 |
| P1 | -0.01609 | 0.708037 | 38 | 0.005863 | 13 | 16.41711 | 6 | 0.038434 | 8 |
| P2 | -0.19772 | 3.37E-06 | 1 | 0.013475 | 2 | 37.12705 | 1 | 0.282771 | 1 |
| P3 | -0.15 | 0.000448 | 2 | 0.008802 | 8 | 7.318244 | 24 | 0.001245 | 28 |
| r1 | 0.101355 | 0.018047 | 10 | -0.00454 | 25 | 20.42425 | 2 | 0.122103 | 2 |
| R1 | -0.01667 | 0.698004 | 37 | -0.00212 | 25 | 0 | 32 | 0 | 32 |
| R2 | 0.086065 | 0.044807 | 14 | -0.01104 | 25 | 7.36983 | 23 | 0.000116 | 29 |
| R3 | 0.113405 | 0.008109 | 5 | 0.012896 | 4 | 6.636591 | 27 | 0.033877 | 11 |
| S1 | 0.095168 | 0.026444 | 11 | 0.012801 | 5 | 3.230957 | 30 | 0.008662 | 20 |
| U1 | -0.02368 | 0.58151 | 32 | -0.00103 | 25 | 0 | 32 | 0 | 32 |
| U2 | 0.032394 | 0.450847 | 28 | 0.00837 | 10 | 0 | 32 | 0 | 32 |
| U3 | 0.019382 | 0.651949 | 36 | 0.000383 | 23 | 0 | 32 | 0 | 32 |
| V1 | 0.038275 | 0.372935 | 25 | 8.63E-05 | 24 | 0 | 32 | 0 | 32 |
| V2 | -0.07971 | 0.063198 | 15 | -0.0119 | 25 | 12.14051 | 16 | 0.006086 | 24 |
| V3 | -0.08954 | 0.036823 | 13 | 0.020533 | 1 | 16.90828 | 5 | 0.056125 | 5 |
| Y1 | -0.00018 | 0.996681 | 45 | -0.00462 | 25 | 7.251745 | 25 | 0.01857 | 13 |
| Y2 | -0.04949 | 0.24916 | 22 | 0.004369 | 15 | 16.02859 | 7 | 0.020654 | 12 |
| Y3 | 0.015955 | 0.710422 | 39 | -0.00794 | 25 | 8.058802 | 21 | 8.48E-08 | 31 |
| Z1 | 0.095112 | 0.026534 | 12 | 0.0089 | 7 | 0 | 32 | 0 | 32 |
| Z2 | 0.005874 | 0.891282 | 42 | -0.00183 | 25 | 0 | 32 | 0 | 32 |
| Z3 | 0.003366 | 0.937569 | 43 | 0.003545 | 18 | 0 | 32 | 0 | 32 |
